# Efficacy and safety of plasma-derived von Willebrand factor/factor VIII concentrate (wilate) prophylaxis in children and adolescents with von Willebrand disease – WIL-31 study post hoc analysis

**DOI:** 10.1016/j.rpth.2025.102719

**Published:** 2025-02-28

**Authors:** Robert F. Sidonio, Leonid Dubey, Kateryna V. Vilchevska, Adlette Inati, Claudia Djambas Khayat

**Affiliations:** 1 Department of Pediatrics, Emory University School of Medicine, Atlanta, Georgia, USA; 2 Communal Nonprofit Enterprise “Western Ukrainian Specialized Children’s Medical Center” of Lviv Regional Council, Lviv, Ukraine; 3 National Specialized Children’s Hospital Okhmatdyt, Center of Hemostasis Pathologies, Kyiv, Ukraine; 4 NINI Hospital and University of Balamand School of Medicine and Medical Sciences, Balamand, Lebanon; 5 Hotel Dieu de France Hospital, Saint Joseph University, Beirut, Lebanon

**Keywords:** adolescent, child, hemostatics, prevention and control, von Willebrand disease, von Willebrand factor

## Abstract

**Background:**

Prophylaxis with von Willebrand factor is recommended in people with severe von Willebrand disease (VWD), regardless of age. WIL-31, the only prospective study with an on-demand run-in study as an intraindividual comparator, demonstrated the efficacy and safety of prophylaxis with the plasma-derived von Willebrand factor/factor VIII concentrate wilate (Octapharma) in adults and children with VWD of all types. Prophylaxis is often considered in young children and adolescents with severe VWD and recurrent bleeding, although limited data support this strategy.

**Objectives:**

To assess the efficacy of wilate prophylaxis in children (6-11 years) and adolescents (12-16 years) in WIL-31.

**Methods:**

Patients received 20 to 40 IU/kg (Octapharma) wilate prophylaxis 2 to 3 times weekly for 12 months. Results were compared with prospective on-demand treatment.

**Results:**

Mean total annualized bleeding rates (ABRs) during on-demand vs prophylaxis were 32.5 vs 3.7 in children (*n* = 9) and 28.9 vs 4.3 in adolescents (*n* = 6), representing reductions of 89% and 85%, respectively. All 34 bleeds in children, and 20/26 (77%) bleeds in adolescents were minor. Mean spontaneous ABRs during prophylaxis were 2.5 in children and 1.5 in adolescents. The most common bleeding site in both groups and across all VWD types was the nose. ABRs were reduced further during the second 6 months of prophylaxis vs the first 6 months. During the second 6 months, 78% of children and 67% of adolescents had zero spontaneous bleeds. No serious adverse events related to study treatment or thrombotic events were observed.

**Conclusion:**

wilate prophylaxis was efficacious and well-tolerated in children and adolescents with all types of VWD.

## Essentials

• Bleed prevention therapy is recommended for VWD patients of all ages.
• The WIL-31 study showed that wilate helps prevent bleeds in people with all types of VWD.
• wilate prophylaxis was effective in children and adolescents with VWD.
• wilate prophylaxis was well tolerated in children and adolescents with VWD.

## 1 Introduction

von Willebrand disease (VWD) is the most common inherited bleeding disorder, affecting approximately 1% of the population [1]. It results from defects in von Willebrand factor (VWF), leading to increased bleeding symptoms [2]. Despite long-term prophylaxis with VWF concentrate being recommended for people with VWD with a history of severe and frequent bleeds regardless of their age [3], it remains underutilized in VWD; less than 10% of people with severe VWD receive VWF prophylaxis [4]. VWD imposes a significant burden on patients, particularly children and adolescents, who may experience disruptions to their education and daily activities [5]. Even bleeds that are not necessarily life-threatening can have a severe impact on a patient’s quality of life. Early initiation of prophylaxis is important for preventing long-term joint damage because even limited joint bleeding can contribute to long-term joint deterioration and increased risk of recurrent bleeding [6]. However, there remains a shortage of evidence surrounding the use of long-term prophylaxis in children and adolescents with VWD. The limited evidence available suggests that long-term prophylaxis in children and adolescents with VWD substantially reduces bleed rates and joint damage [7,8].

wilate (Octapharma) is a plasma-derived factor concentrate containing VWF (pdVWF) and factor (F)VIII in a physiological 1:1 activity ratio and is indicated in VWD patients 6 years of age and older for the prevention and treatment of bleeds as well as perioperative management of bleeding [9]. The prospective, international, multicenter WIL-31 study (NCT04052698) demonstrated the efficacy of wilate prophylaxis in adults and children with VWD of all types. The primary endpoint of >50% reduction in mean total annualized bleeding rate (ABR) was met, with an 84% reduction observed [10]. The objective of this post hoc analysis was to investigate the efficacy and safety of regular prophylaxis with wilate vs prior on-demand treatment, specifically in children and adolescents (6-16 years of age) with VWD.

## 2 Methods

### 2.1 Study design

WIL-31 (NCT04052698) was a prospective, noncontrolled, international, multicenter phase 3 trial that enrolled male/female patients aged ≥6 years with VWD type 1 (VWF ristocetin cofactor < 30 IU/dL), type 2 (except 2N), or type 3. Prior to entering the WIL-31 study, all patients had received on-demand treatment with a pdVWF/FVIII concentrate during a 6-month prospective, observational run-in study (WIL-29); patients who experienced at least 6 bleeding episodes (BEs) during WIL-29, excluding menstrual bleeds, with at least 2 of these BEs treated with a VWF-containing product, were eligible to enter WIL-31. Patients in WIL-31 received wilate prophylaxis 2 to 3 times per week at a dose of 20 to 40 IU/kg for 12 months. The prophylactic dose for each patient was determined by the principal investigator, based on each patient’s clinical condition, and could be adapted based on individual patient responses. FVIII and VWF activity levels were measured in all patients at baseline and each study visit (occurring after 1, 2, 3, 6, 9, and 12 months of treatment) using the VWF ristocetin cofactor assay and for FVIII by the chromogenic and one-stage assays. Target joints were defined as having 3 or more spontaneous BEs in a single joint within 6 consecutive months. The study was performed in accordance with the Declaration of Helsinki and the respective local regulations. Voluntarily given, informed consent was obtained from patients (or their legal guardians) before any study-related procedures were conducted. For further details, please refer to the primary publication [10].

### 2.2 Statistical analysis

Post hoc analyses were performed on data from children (6-11 years old) and adolescents (12-16 years old) with confirmed VWD who completed WIL-31. Total, spontaneous, and site ABRs and VWF and FVIII levels were assessed. All statistical analyses were descriptive.

## 3 Results

### 3.1 Patient disposition

Of the 33 patients with confirmed VWD who completed the WIL-31 study, 9 (27%) were children, and 6 (18%) were adolescents. The demographics and baseline characteristics of the children and adolescents are summarized in Table 1. All VWD types (except 2N) were represented in both age groups, with the majority of patients (9/15; 60%) having type 3 VWD.

**Table 1:** Demographics and baseline characteristics of children (6-11 years) and adolescents (12-16 years) with severe von Willebrand disease included in the analysis.

Characteristic	Children	Adolescents
	(*n* = 9)	(*n* = 6)
Gender, *n* (%)		
Male	6 (66.6)	3 (50.0)
Female	3 (33.3)	3 (50.0)
VWD type, *n* (%)		
Type 1	2 (22.2)	1 (16.7)
Type 2A	2 (22.2)	1 (16.7)
Type 3	5 (55.6)	4 (66.7)
Age (y) at screening, median (range)	8.9 (7-11)	14.5 (12-16)
Weight (kg) at screening, median (range)	27.5 (20.5-41.0)	67.0 (39.9-92.0)
Height (cm) at screening, median (range)	136 (118-146)	165 (151-182)
Race, *n* (%)		
Caucasian	9 (100)	6 (100)
Blood type, *n* (%)		
A	7 (77.8)	2 (33.3)
B	1 (11.1)	1 (16.7)
AB	1 (11.1)	0 (0.0)
O	0 (0.0)	3 (50.0)
Family history of VWD, *n* (%)	4 (44.4)	4 (66.7)
FVIII and VWF activity, IU/dL, mean (SD)		
FVIII:chromogenic assay	14.6 (14.2)	6.3 (4.6)
FVIII:one-stage assay	17.0 (16.2)	8.8 (9.2)
VWF:Rco assay	6.8 (2.9)	5.4 (0.9)

FVIII, factor VIII; VWD, von Willebrand disease; VWF, von Willebrand factor; VWF:Rco, VWF ristocetin cofactor.

### 3.2 Overview of BEs

**Figure 1:**
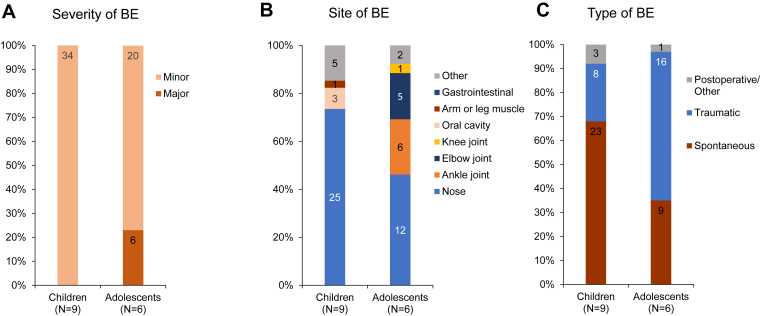
Severity (A), site (B), and type (C) of bleeding episodes (BEs) in children and adolescents with severe von Willebrand disease during wilate prophylaxis. “Other” bleeds in children during WIL-31 included cutaneous, head, hip, and toe; “Other” bleeds in adolescents during WIL-31 included cutaneous and tonsil.

During 12 months of prophylaxis, 67% (6/9) of children and 83% (5/6) of adolescents experienced 34 and 26 bleeds, respectively. All the bleeds in the children were minor. In adolescents, 20 of the 26 (77%) bleeds were minor, and 6 (23%) were major (Figure 1A). The most common bleeding sites were the nose (74%; 25/34) and oral cavity (9%; 3/34) for children and the nose (46%; 12/26), ankle (23%; 6/26), and elbow (19%; 5/26) joints for adolescents (Figure 1B). In children, 68% (23/34) of bleeds were spontaneous, 24% (8/34) were traumatic, and 9% (3/34) were due to allergic rhinitis. In adolescents, 35% (9/26) of bleeds were spontaneous, 62% (16/26) were traumatic, and 1 was a postinfection bleed (Figure 1C). For children and adolescents, 82% (28/34) and 77% (20/26) of bleeds required treatment, with “excellent/good” efficacy achieved in 100% and 95% of treated bleeds, respectively.

### 3.3 ABRs

**Figure 2:**
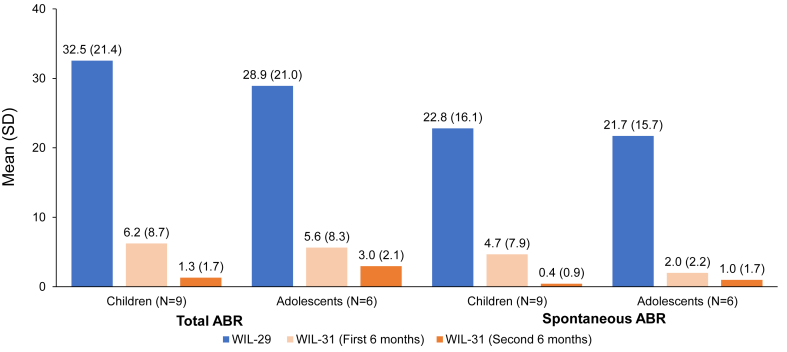
Mean total annualized bleeding rate (ABR) and spontaneous ABR for severe von Willebrand disease patients in WIL-29 and WIL-31 by the 6-month period.

The mean total ABRs during on-demand vs prophylaxis were 32.5 vs 3.7 in children and 28.9 vs 4.3 in adolescents, representing 89% and 85% reductions during prophylaxis compared with on-demand treatment, respectively (Table 2). The mean total ABRs were 6.2 and 1.3 in the first and second 6-month periods of prophylaxis in children, respectively, and 5.6 and 3.0 in adolescents, respectively (Figure 2).

**Table 2:** Annualized bleeding rates during on-demand treatment with von Willebrand factor-containing products (WIL-29) and during prophylaxis (WIL-31).

ABR	Age group					
	Children			Adolescents		
	WIL-29	WIL-31	Reduction (%)	WIL-29	WIL-31	Reduction (%)
Total						
Mean	32.5	3.7	88.5	28.9	4.3	85.2
Median	24.0	1.0	95.9	22.5	3.4	84.7
Spontaneous						
Mean	22.8	2.5	89.0	21.7	1.5	93.1
Median	24.0	1.0	95.9	22.5	1.0	95.7
Total treated						
Mean	30.9	3.1	90.1	22.0	3.3	85.0
Median	24.0	1.0	95.9	18.4	3.4	81.3
Spontaneous treated						
Mean	21.5	2.0	90.8	18.7	1.5	92.0
Median	18.9	0.0	100.0	18.4	1.0	94.7

ABR, annualized bleeding rate.

**Figure 3:**
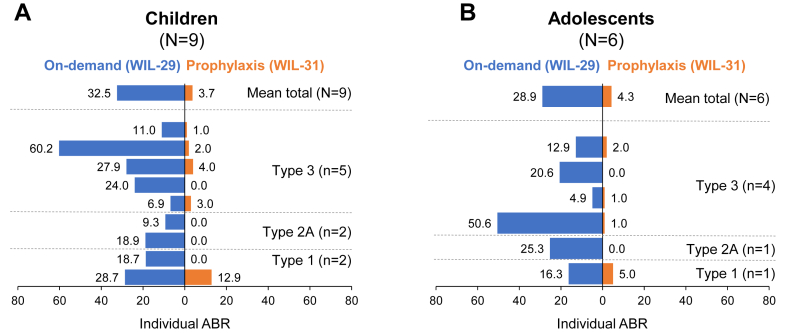
Individual spontaneous annualized bleeding rates (ABRs) in children (A) and adolescents (B) with severe von Willebrand disease during WIL-29 and WIL-31.

The mean spontaneous ABRs during on-demand treatment vs prophylaxis were 22.8 vs 2.5 (89% reduction) in children and 21.7 vs 1.5 (93% reduction) in adolescents (Figure 3). During the first and second 6 months of prophylaxis, children had spontaneous mean ABRs of 4.7 and 0.4, respectively, while adolescents had mean ABRs of 2.0 and 1.0, respectively (Figure 2).

During 12 months of prophylaxis, 44% of children and 33% of adolescents had zero spontaneous bleeding events. In the first 6 months of prophylaxis, 33% of children and 56% of adolescents had zero spontaneous bleeding events compared with 78% and 67% during the second 6 months, respectively. Every child and adolescent had at least 1 spontaneous bleeding event during 6 months of on-demand treatment.

**Figure 4:**
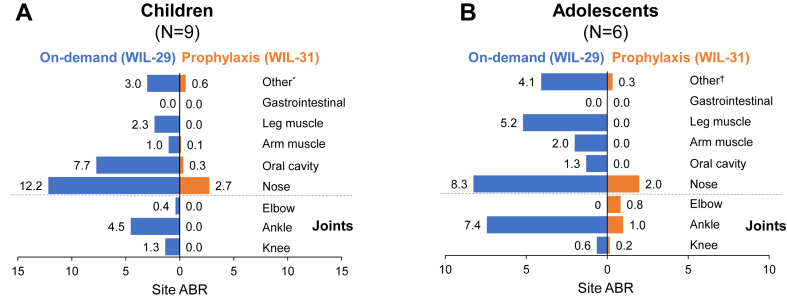
Total annualized bleeding rate (ABR) by bleeding site in children (A) and adolescents (B) with severe von Willebrand disease during WIL-29 and WIL-31. ∗“Other” bleeds in children during WIL-29 include ecchymosis, hand, hip, shoulder, and toe; “Other” bleeds in children during WIL-31 include cutaneous, head, hip, and toe. †“Other” bleeds in adolescents during WIL-29 include cutaneous, hand, subcutaneous, and toe; “Other bleeds in adolescents during WIL-31 include cutaneous and tonsil.

During prophylaxis, children experienced reduced ABRs at all sites compared with during on-demand treatment. Adolescents experienced reduced ABRs at all nonjoint sites compared with on-demand treatment (Figure 4).

### 3.4 Joint bleeds

The joint ABR was 6.5 in children during on-demand treatment compared with 0.1 during prophylaxis (98% reduction). Only 1 joint bleed occurred in children during WIL-31, a traumatic bleed affecting the right hip joint. The mean joint ABRs during on-demand treatment vs prophylaxis were 1.3 vs 0.0 (100% reduction) for knees, 4.5 vs 0.0 (100% reduction) for ankles, and 0.4 vs 0.0 (100% reduction) for elbows.

The joint ABR was 8.9 in adolescents during on-demand treatment compared with 2.0 during prophylaxis (78% reduction). The mean ABRs during on-demand treatment vs prophylaxis were 0.6 vs 0.2 (74% reduction) for knees, 7.4 vs 1.0 (87% reduction) for ankles, and 0.0 vs 0.8 for elbows. The elbow joint bleeds all occurred in a 15-year-old type 3 VWD patient who already had arthropathy in the elbow at the start of WIL-29. Four of the bleeds during WIL-31 were traumatic minor bleeds, and the other was a spontaneous major bleed. All 5 bleeds during WIL-31 were treated with a single injection of wilate.

At the start of WIL-31, 2 patients with type 3 VWD (aged 8 and 16 years) had a target joint (right ankle joint in both patients) with 12 bleeds and 15 bleeds during the 6 months of on-demand treatment, respectively. Both joints had resolved by the end of the WIL-31 study (12 months of prophylaxis). The child’s target joint had no bleeds over the 12 months of prophylaxis. The adolescent’s target joint had 5 traumatic bleeds and 1 spontaneous bleed during the 12 months of prophylaxis.

### 3.5 wilate dosing

Children received a median (range) dose of 32.9 (21.2-39.6) IU/kg per injection, and adolescents 35.9 (28.4-38.9) IU/kg. Children received a median (range) weekly dose of 66.3 (42.6-112.5) IU/kg wilate during prophylaxis, and adolescents 64.4 (52.6-101.0) IU/kg. The median (range) weekly dose of wilate for prophylaxis in the overall study population (including adults) was 58.3 (28.2-113.7) IU/kg.

Six children (67%) were treated twice weekly for the entire study, 1 child was treated 3 times weekly, and 2 children were switched from twice to 3 times weekly during the study. Four adolescents (67%) were treated twice weekly throughout the study, and 2 were switched from twice to 3 times weekly during the study.

### 3.6 Safety and tolerability

No serious adverse events related to the study treatment and no thrombotic events were observed in children or adolescents in WIL-31. FVIII/VWF activity data showed that there was no accumulation of VWF or FVIII during the 12 months of prophylaxis in children or adolescents. No treatment-emergent adverse events resulted in the discontinuation of the study medication in children or adolescents. There were no safety concerns raised by the clinical laboratory, vital signs, or physical examination findings. There were no cases of parvovirus B19 seroconversion or inhibitor development judged to be related to study treatment. Two brothers were found to have VWF inhibitors during WIL-31, which were subsequently found to have already been present prior to the start of the study (Khayat et al., manuscript in preparation).

## 4 Discussion

WIL-31 is the largest prospective study specifically investigating the efficacy and safety of VWF prophylaxis in patients with VWD. Prophylaxis with wilate was efficacious and well-tolerated in people with all types of VWD [10]. Here, we report that prophylaxis reduced the mean total ABR by 89% and 85% in children and adolescents, respectively, compared with prior on-demand treatment with any pdVWF/FVIII concentrate. These results are consistent with the results in the overall study population, where the total ABR was reduced by 84% [10]. Furthermore, as the majority of patients in this analysis (60%) had type 3 VWD, which is typically associated with severe bleeding and complications [11], these results demonstrate wilate’s efficacy across the most severe disease types. ABRs were further reduced during the second 6 months of prophylaxis compared with the first 6 months, providing data on the short-term benefit of VWF concentrate prophylaxis in this population. No serious treatment-emergent adverse events or thrombotic events were reported during the 12 months of wilate prophylaxis.

The results of the WIL-31 study are consistent with previously published data on wilate prophylaxis in children and adolescents. In an analysis pooling data from 4 phase 3 trials, the monthly bleeding rate in 4 children under 12 years of age who received prophylaxis with wilate for 11.5 to 46.0 months reduced from 3.4 while on prior on-demand treatment to 1.5 on prophylaxis [12]. In this pooled dataset, another 2 children who switched from prophylaxis with the pdVWF/FVIII concentrate Haemate-P (CSL Behring) to prophylaxis with wilate also experienced reductions in bleeding rates [12]. Similarly, in an analysis of real-life data, 3 children with joint bleeding and 1 child with recurrent gastrointestinal bleeding were given wilate. No breakthrough bleeds were observed in 3 of the 4 children during the observation period [13].

No children under 6 years of age were included in WIL-31. However, in a previous study of 15 children under 6, the efficacy of wilate was rated as excellent or good for 99.7% of prophylaxis infusions [14]. Furthermore, the ongoing phase 3 WIL-33 study aims to determine the efficacy, pharmacokinetics, immunogenicity, and safety of wilate as routine prophylaxis for 12 months, specifically in children under the age of 6 years with severe VWD, and primary completion is expected in December 2024 [15].

Despite the importance of bleed prevention in children and adolescents with VWD, published data on the efficacy of prophylaxis in these populations are very limited. A number of studies have included children and adolescents but have not specifically reported the outcomes of these subpopulations [[16], [17], [18], [19], [20], [21]]. A study from the VWD Prophylaxis Network found that prophylaxis significantly reduced the median intraindividual number of bleeds compared with prior treatment for 26 children and adolescents and 33 adults (*P* <.0001), but the authors did not provide specific details for the children or adolescents [22]. A further study from the VWD Prophylaxis Network included 49 children. Epistaxis was the most common reason in children for initiating prophylaxis, but the response to prophylaxis was not reported in this population [23]. During the studies with von Willebrand factor/Factor VIII-von Willebrand disease (SWIFTLY-VWD) trial [24], 4 children who received prophylaxis with the pdVWF/FVIII concentrate Voncento (CSL Behring) experienced a median of 23.5 nonsurgical BEs during 12 to 13 months of prophylaxis. Although the number of BEs during prophylaxis was compared with those during prior on-demand treatment, the latter were not collected prospectively (as they were during the WIL-29 run-in study). Three of the children from the SWIFTLY-VWD trial were followed for up to 34 months [25], and they experienced a median of 22 nonsurgical BEs per year. Of 48 patients with VWD treated in Sweden, 12 children began prophylaxis with Haemate-P before the age of 5 years, and 7 began prophylaxis between the ages of 5 and 15 years. There was a substantial reduction in BEs compared with prior treatment. Among the children who began prophylaxis before age 5 for the prevention of nose and mouth bleeds, none reported joint bleeds or had signs of clinical arthropathy [7]. In a study of 13 children and 7 adolescents with VWD receiving prophylaxis with Haemate-P or wilate, recurrent BEs stopped in all of the patients except for one 7-year-old patient with type 3 VWD who developed VWF inhibitors after 48 exposure days to Haemate-P [8].

A small number of case reports have also been published reporting on prophylaxis in young children. For example, Gouider et al. [26] recently reported the case of a 2-year-old child with type 3 VWD who experienced multiple bruises and traumatic bleeding of soft tissues, which improved on prophylaxis with the pdVWF Wilfactin (LFB Biopharmaceuticals). Another case report described a child with type 3 VWD who had received prophylaxis with Wilfactin in combination with FVIII concentrate (Wilstart/Factane [LFB Biopharmaceuticals]) from 38 months of age [27]. Because of a high bleeding rate, the child was switched during late childhood to wilate, and the total ABR was reduced by 73% compared with prophylaxis with Wilfactin. The patient’s mother also reported a marked increase in quality of life for herself and her son.

Managing VWD in children and adolescents is complex due to several factors. First, the efficacy of prophylactic treatment for VWD in this population is not well-established. Second, early diagnosis of VWD in childhood can be challenging, as bleeding symptoms often do not correlate strongly with VWF levels or activity [28]. Mucocutaneous bleeding, bruising, and oropharyngeal bleeding are common in both children with and without VWD, further complicating the diagnosis [29]. Additionally, children with VWD may remain asymptomatic, especially if they have not experienced significant hemostatic challenges [28]. To ensure timely diagnosis and appropriate treatment, it is crucial to raise awareness of VWD signs and symptoms among healthcare professionals and caregivers.

Prophylaxis with VWF concentrates is recommended for patients with VWD with a history of severe and frequent bleeds, regardless of their age [3]. The success of early initiation of prophylaxis in people with hemophilia A, which has notably reduced bleeding rates and enhanced patients’ quality of life while preventing long-term joint damage, is a compelling rationale for employing a similar approach for people with VWD [4]. In the Willebrand in the Netherlands study, 23% of patients with VWD reported joint bleeding, with 65% of these patients experiencing their first joint bleed before 16 years of age [6]. Joint bleeds can lead to restrictions on physical activity, even in children [28], and repeated joint bleeding is a major risk for the development of arthropathy. Preventing joint bleeds and resolving target joints are, therefore, important to preserve long-term joint health [30]. During WIL-31, joint ABRs were reduced by 98% for children and 78% for adolescents. In both patients with target joints at the start of the study, the target joints had resolved by the end of the study, and no patient developed a new target joint. These data suggest that wilate prophylaxis may be an interesting approach to reducing the risk of arthropathy and long-term immobility in children and adolescents with VWD.

No safety concerns were identified in children or adolescents throughout the 12-month period of prophylaxis. This observation is consistent with findings from other studies investigating the use of VWF concentrates in pediatric and adolescent populations, indicating a generally well-tolerated profile of VWF concentrate use in these age groups [[24], [25], [26], [27]]. Although inhibitor development has been reported in patients treated with VWF concentrates, it is much less commonly observed than inhibitor development in hemophilia [8,31]. During WIL-31, it was discovered that 2 brothers in the study already had inhibitors prior to the start of the study. Despite the presence of inhibitors, wilate prophylaxis reduced the bleeding rate in both patients, and the inhibitors resolved by the end of the study in 1 case (Khayat 2025, manuscript in preparation).

Limitations of the WIL-31 study are that it was open-label, not randomized or controlled, included mostly Caucasian patients, and had no patients with type 2N VWD. Furthermore, the group sizes used in the subanalyses were relatively small.

## 5 Conclusions

Data on VWF prophylaxis in children and adolescents with VWD are scarce and imprecise. WIL-31 stands out as the largest study of VWF prophylaxis in this population and the only one incorporating an on-demand run-in study as an intraindividual patient comparator. Prophylaxis with wilate was efficacious and well-tolerated in children and adolescents with all types of VWD, with substantial reductions in bleeding rates compared with prior on-demand treatment. The study provides much-needed data on the efficacy of prophylaxis in VWD and supports the use of prophylaxis for children and adolescents with a severe bleeding phenotype.
